# Gut-associated lymphoid tissue: a microbiota-driven hub of B cell immunity

**DOI:** 10.1016/j.it.2024.01.006

**Published:** 2024-03

**Authors:** Mats Bemark, Michael J. Pitcher, Chiara Dionisi, Jo Spencer

**Affiliations:** 1 Department of Translational Medicine – Human Immunology, Lund University, J Waldenströms gata 35, Malmö, Sweden; 2 Department of Clinical Immunology and Transfusion Medicine, Sahlgrenska University Hospital, Region Västra Götaland, Gothenburg, Sweden; 3 Peter Gorer Department of Immunobiology, King’s College London, Guy’s Hospital Campus, St Thomas’ Street, London SE1 9RT, UK

## Abstract

The diverse gut microbiota, which is associated with mucosal health and general wellbeing, maintains gut-associated lymphoid tissues (GALT) in a chronically activated state, including sustainment of germinal centers in a context of high antigenic load. This influences the rules for B cell engagement with antigen and the potential consequences. Recent data have highlighted differences between GALT and other lymphoid tissues. For example, GALT propagates IgA responses against glycans that show signs of having been generated in germinal centers. Other findings suggest that humans are among those species where GALT supports the diversification, propagation, and possibly selection of systemic B cells. Here, we review novel findings that identify GALT as distinctive, and able to support these processes.

## Highlights

Mammalian gut-associated lymphoid tissue (GALT) is chronically activated by the intestinal microbiota throughout life.
GALT propagates and selects B cells in germinal centers, including B cells recognizing T-cell-independent carbohydrate antigens.
GALT supports the development of systemic B cells in the so-called GALT species, including humans.
In humans, GALT supports the development of innate-like marginal zone B cells that circulate in blood, mostly reside in the spleen, and can protect the lungs.

## Significance

Gut-associated lymphoid tissue (GALT) is located throughout the gastrointestinal tract and is indispensable for the maintenance of gut homeostasis and human health. Recent advances increase our understanding of B cell responses to microbiota in GALT that can generate antibodies to antigens often shared by microbiota species, including T cell independent, yet germinal-center-matured responses. Indeed, chronic stimulation of GALT B cells generates a niche for B cell maturation and propagation in some species, including marginal zone B cells in humans.

## GALT in intestinal and systemic immunity

**Figure 1:**
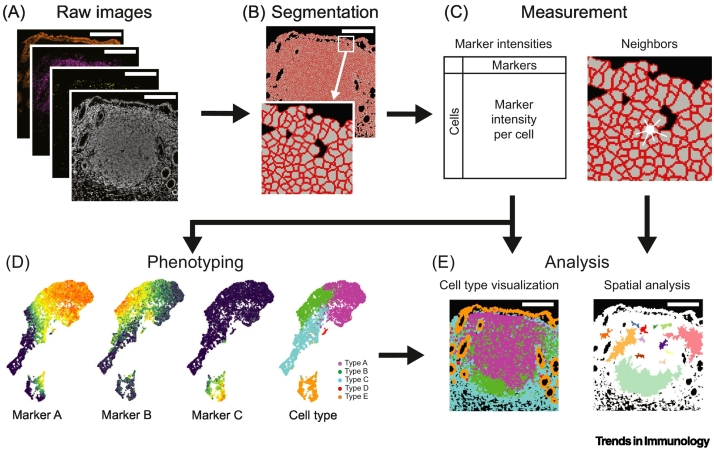
Example pipeline for processing image data. An example pipeline for processing image data using an imaging mass cytometry example is shown. (A) Raw image files for each region of interest (ROI) are extracted, including cell membrane markers and DNA interlocutor. (B) ROIs are segmented using the image files from (A) to determine cell boundaries within the ROI. (C) Cell measurements are taken including the marker intensity for each cell [from images in (A) and cell boundaries in B)] and cell neighbors based on cell–cell proximities. (D) Cells are assigned identities based on their marker intensities from (C) using, for example, dimensionality reduction and clustering based on key markers. (E) Further analysis is performed, for example visualization of computationally derived cell types back on ROIs and neighborhood analysis finding patches containing similar cell–cell interactions based on assigned phenotypes from (D) and neighbors from (C).

The importance of **gut-associated lymphoid tissue (GALT)** (see Glossary) for the maintenance of health has been known for decades [1]. Despite its small size relative to that of the gastrointestinal tract, GALT maintains homeostasis by sustained generation of immune effector cells that disseminate through the full length of the gut [1]. Here, we discuss the unique features of GALT and its interactions with microbiota that support the production of distinctive antibody specificities, and how the same niche can support the development of subsets of systemic B cells in some species, including mediators of rapid systemic immunity to **T-independent (TI)** antigens in humans [2]. Progress in this field is being enhanced by the availability of technologies allowing in depth-analysis of small and often inaccessible tissues (Box 1 and Figure 1).

Box 1 **Multiplexed technologies can enrich our understanding of GALT**

**Single cell transcriptomics**

Functional studies of human GALT have been confounded by the small size of the regions of interest and difficulty in accessing tissues that can be invisible to the naked eye in humans. However, recent advancements in technologies and analytical methods have helped to overcome some of these challenges. Single-cell transcriptomics methods, such as scRNA-seq, have allowed unbiased characterization of immune cells within GALT of mice and humans [73]. Inclusion of antibody variable region gene sequencing for each cell allows the analysis of clonal relationships and has been used to identify clonal dissemination between GALT and other lymphoid organs [62].

**Cell surface imaging**

Recent and ongoing advancements in imaging techniques enable viewing of the spatial GALT structure. Imaging mass cytometry (IMC) exploits cytometry by **time-of-flight techniques** such as CyTOF and imaging mass cytometry, allowing the digital capture of more than 40 antibody-labeled proteins in tissue sections; it has been utilized to computationally dissect lymphoid tissue structures (see Figure 1 in main text). It has also been applied to study appendiceal dysbiosis in the context of human pancreatic cancer [74], visualize the development of human fetal intestinal immunity [75], as well as profile and localize B cell subsets in human GALT [18].

**RNAScope**

IMC can also be supported by the analysis of gene expression using the RNAScope *in situ* hybridization assay to detect specific RNA [75]. This technique has profiled the infection of epithelial cells in the human small intestine by norovirus [76], and cells in the gut infected by SIV in nonhuman primates [77]. By integrating RNAScope methods into IMC panels to confirm low resolution spatial transcriptomics at a single cell level, we have visualized the expression of the SLE autoantigen DNASE1L3 by LysoDC in the microanatomical context of the GALT SED in humans; the preliminary results which remain to be validated show contrasts in the distribution of DNASE1L3 in mice [18].

**Spatial transcriptomics**

Even when combined with RNAScope, imaging techniques such as IMC are limited in the number of markers they can include. They fail to capture the vast transcriptomic heterogeneity within lymphoid cells and the potential associations of immune cells with the stroma. Spatial transcriptomics combines the high dimensionality of scRNA-seq with the spatial insights gained from imaging techniques [78]. This can incorporate spatial information of B and T cell receptor clone signatures. These technologies allow the capture of genes at a resolution of ~55 μm, with each spot capturing 1–10 cells depending on cell size and location [79]. Advancements in resolution that allow the spatial capture of gene expression in single cells have been used to study cellular heterogeneity and location in the healthy intestine and in inflammatory bowel disease in humans [80,81]. We anticipate that increased availability of such methods and further refinement of computational platforms on which to analyze data may propel the next generation of advances in this field.

Alt-text: Box 1

## GALT and antigen sampling

Mammalian GALT is organized lymphoid tissue that sits in foci adjacent to the epithelium throughout the gut. In many species, including humans, it is most abundant in the **Peyer’s patches (PPs)** of the terminal ileum [1,3,4]. Structurally, GALT superficially resembles lymph nodes and palatine tonsils. All have B cell follicles and associated T cell zones containing high endothelial venules through which blood lymphocytes can extravasate and segregate along chemokine gradients to form B cell and T cell zones. However, unlike lymph nodes, GALT and palatine tonsils have no capsule and no afferent lymphatics and thus, the way antigens enter and are detected varies [4., 5., 6.]. GALT differs from palatine tonsils in the structure of the epithelial boundary between the lymphoid tissue and the external tissues of the body. While this boundary is pseudostratified in palatine tonsils [5], it is a single-cell-thick barrier in GALT termed the **follicle-associated epithelium (FAE)** that includes antigen-sampling **microfold (M) cells** [7]. Also, in terms of cell traffic, human GALT recruits cells expressing the α4β7 integrin via the mucosal endothelial addressin MAdCAM1 [8,9], while the palatine tonsils do not – the latter being more dependent on recruitment via the peripheral node addressin (PNAd) than the former [5,10].

Lymphatics that drain nonmucosal barrier sites such as skin deliver antigen-loaded dendritic cells (DCs) as well as free antigen into lymph nodes via afferent lymphatics that drain into the subcapsular sinus [6]. However, as long as there is no breach of the skin barrier, no foreign antigens are transferred and thus, peripheral lymph nodes are usually sterile and immunologically inactive [6]. Similarly, the spleen that encounters antigens present in blood will mostly be sterile and quiescent [11]. In contrast, GALT, palatine tonsil, and lymph nodes that drain mucosal surfaces will constantly encounter foreign structures from commensal microbiota and infective pathogens, as well as ingested or inhaled antigens. This results in constant encounters between immune cells and antigens, resulting in ongoing immune responses including the formation of **germinal centers (GCs)** [12] that shape the commensal microbiota as well as the responses to pathogen invasion [7,13].

## The unique cellular composition of the subepithelial dome

Antigen sampled from the gut lumen via the FAE, either by M cells or DCs that extend dendrites through the FAE, arrives in the **subepithelial dome region (SED)** of the follicle [4,14]. In addition to B cells and sparse T cells, the SED includes several myeloid cell populations, including classical DCs (cDCs) and macrophages, as well as cells termed LysoMacs and **LysoDCs** containing microbicides such as lysozyme and NOX2 that can kill bacteria and similarly to cDCs can present antigen to T cells [7,12,15., 16., 17.]. Preliminary data which remains to be validated indicates that they also express the enzyme DNASE1L3 that ensures that pathogenic microbial DNA is also removed (in preprint) [18]. Thus, LysoDCs potentially ensure that live bacteria sampled from the gut lumen, including shared self-antigens such as DNA that may help trigger B cell activation thorough binding to **Toll-like receptors (TLRs),** do not persist in the host below this cellular and enzymatic barrier [17,19].

**Figure 2:**
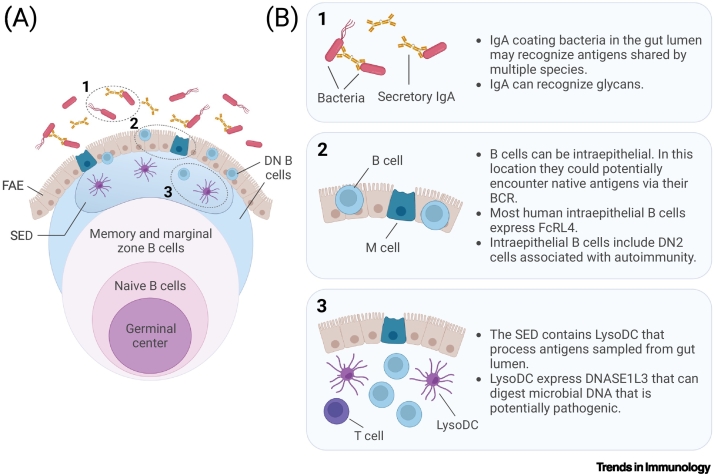
Key figure. Layering of B cells in human gut-associated lymphoid tissue (GALT) with a focus on the subepithelial dome (SED). Schematic representation of human GALT (A) identifying the positions of B cell subsets relative to other key contributors to the follicle-associated epithelium (FAE), which forms the boundary between the microbiota and the host. The germinal center (GC) is a ubiquitous feature of lymphoid tissue in GALT. Surrounding the GC is a zone of naive B cells. This in turn is encircled by the marginal zone and memory B cells. B cells have also been detected in the epithelium [20], where they show expression of Fc receptor-like 4 (FcRL4) [23]. Most B cells adjacent to and within the FAE are double negative (DN) B cells, predominantly of the DN2 subtype, which can accumulate in certain inflammatory and autoimmune diseases [18,25,31,84]. Specialized LysoDCs are present in the SED, where they intermingle with mostly B cells, and handle microbes that are actively sampled by microfold (M) cells in the FAE from the gut lumen [15,17]. LysoDCs contain microbicides and can digest bacterial DNA [16,18]. Above the FAE, secretory IgA-coating bacteria in the gut lumen recognize antigens shared by different species [50], including glycans such as the LPS-O antigen [51]. Areas numbered 1–3 in (A) are illustrated in more detail in (B), along with a description of features that are relevant to this review. This figure was created using Biorender.com.

Where and how B cells in GALT encounter antigens and the consequences of the encounter are important to consider because this will shape the B cell response and the ability of the cell to present antigen peptides to T cells. As B cells interact with the external surfaces of antigens through their B cell receptor (BCR), they need to encounter intact antigen. Bacteria can indeed be detected within the SED where they exist alongside DC subsets, able to kill them and dispose of the debris [17,18]. It would therefore seem logical to presume that B cell encounters with antigens from the gut lumen should occur early and before bacteria reach the barrier of microbicidal LysoDCs. B cells are present on the front line of SED, including within the epithelium where they could logically encounter native antigen [20,21] (Figure 2, Key figure).

The majority of human B cells in the FAE express **Fc receptor-like 4 (FcRL4)** that is a member of a family of Ig domain-containing type I membrane proteins (FcRL1–5) [22., 23., 24.]. Preliminary evidence that remains to be validated suggests that most FcRL4^+^ cells lack or have low expression of the classical marker of memory B cells CD27 and IgD (which is highly expressed in naive B cells) and can therefore be classified as double negative (DN) [18]. DN cells can be divided into DN1 and DN2 cells according to phenotype. DN2 cells have higher expression of CD20 and CD11c, but lower CD21, relative to DN1 cells [25]. Preliminary data suggest that B cells that express FcRL4 and infiltrate the FAE are DN2 [18]. DN2 cells have been reported to accumulate in certain inflammatory and autoimmune diseases such as systemic lupus erythematosus (SLE), and their numbers are thought to increase with age [25., 26., 27., 28., 29., 30.]. A study identifying FcRL4^+^ B cells in salivary gland ductal epithelium considered the possibility that these cells are components of a proinflammatory response [31]. However, the identification of similar DN2 B cells in normal human GALT epithelium suggests that, although preliminary, this may be normal for B cells infiltrating the mucosal epithelium and is not necessarily a disease-associated feature [18].

FcRL4^+^ cells in GALT are located in a region of antigenic load that is derived from the gut lumen. The protein can act as a low affinity receptor for aggregated IgA [28]. However, FcRL4 harbors 3 tyrosine-based inhibitory motifs (ITIMs) in its intracellular domain which suggests that it may prevent activation rather than drive a response [32]. Nevertheless, FcRL4-expressing B cells can undergo isotype switching (class switch recombination) to IgA, and express markers of activation, including CD69, CD80 and CD86. With a potential to present antigen, these cells may be active participants in the local immune response [27,28].

Thus, B cells in SED of human GALT have unique features that have been associated with autoinflammatory conditions but are not normally abundant in nonmucosal lymphoid tissues.

## Spatial dynamics of B cell encounters with antigen in GALT

The spatial dynamics of B cell/antigen interactions in the SED has almost exclusively been studied in animal models (e.g., mice). Such studies have been limited to few antigens, with the response to **cholera toxin (CT)** studied in greatest detail. Unlike many other antigens, CT is an efficient mucosal antigen with adjuvant properties even when delivered orally [33]. It allows studies of the well-characterized response to the 4-hydroxy-3-nitrophenyl acetyl (NP) hapten in a mucosal context when NP and CT are conjugated and given orally to C57BL/6 mice [34]. This has allowed tracking of transferred naive GFP-expressing NP-specific B cells after immunization. Using fluorescent microscopy and live imaging techniques, a coherent picture has now emerged of B cell activation by NP–CT [21,35].

The first site where activated naive B cells can be detected in mouse PPs after *de novo* oral immunization with NP–CT is in the SED region, just below the M cells [21,36]. Naive B cells that enter PPs migrate to the SED via expression of CCR6 [37]. If antigen is encountered, the B cells will be activated in SED, where they undergo their initial proliferative phase, and also complete IgA class switch recombination (CSR) [35]. *In vivo* depletion experiments have demonstrated that lymphotoxin-β-receptor-positive (LTβR^+^) CD11b^+^ cDC and/or LysoDC are important for IgA CSR, because they express αvβ8 that activates transforming growth factor (TGF)β [36,38]. While it is clear that TGFβ is required for IgA CSR, it is less clear which cells provide the latent protein [37,39,40]. Activated B cells from these early foci subsequently infiltrate pre-existing GCs driven by responses to other antigens [33]. While the early SED proliferation is not highly dependent on affinity, the subsequent entry into the GC is, resulting in an early stage of affinity selection. This may be important because it could contribute to the affinity maturation observed against TI antigens in PPs (see below) [35].

Where cognate B–T cell interactions first occur during the initiation of T-cell-dependent (TD) PP IgA responses is still unclear. B cells recognizing NP–CT interact with T cells in the SED in mice, but B and T cells have been suggested to interact even before entering the SED [22,35,36]. **T follicular helper (Tfh) cells** in GALT have also been proposed to support IgA responses via cross-differentiation of CD4^+^ **regulatory T (Treg) cells** or **T helper (Th)17 cells** [41,42]. This view was based on data from transfers of Treg or Th17 cells into T-cell-deficient mice and, in the case of Th17, an IL-17 expression lineage-tracing strain. Notably, this suggestion comes with a major caveat; since both Treg and Th17 cells form as a consequence of interactions with specific antigens, if Tfh cells are developed from them, in theory, they would only show reactivity to antigens that were previously encountered. Hence, if this was the only possible pathway of generating Tfh cells, TD responses would only occur to antigens that were experienced earlier. We have published data supporting an alternative view [43]. Specifically, using a different mouse transfer model, we found that while natural thymic-derived Treg cells are needed for IgA production, they do not crossdifferentiate into Tfh cells, but instead appear to have an antigen-independent role by providing the TGFβ that is needed for IgA CSR [39]. Based on TCR cloning and single-cell RNA sequencing (scRNA-seq) from wild-type (WT) mice, we showed that Treg and Th17 cells rarely carry the same TCRs as Tfh cells in PPs, either in total T cells or CT-specific cells, which would have been the case if crossdifferentiation occurred during the response [34]. The number of T cells that were labeled due to current or previous expression of IL-17 increased after immunization. However, these Th17 cells did not become Tfh cells, or bind to the same immunodominant peptide/MHC II complex as the Tfh cells did, again arguing against crossdifferentiation [34]. In addition, when RORγt, a transcription factor crucial for Th17 development, was depleted from CD4^+^ T cells using a Cre system, normal numbers of IgA-producing plasma cells were found in the gut, and mice responded with strong antigen-specific serum and intestinal IgA responses, suggesting that Th17 were not needed for normal IgA production, neither during steady-state nor after oral immunization [34]. Thus, although we cannot exclude the possibility that Th17 cells may sometimes crossdifferentiate into Tfh cells to support IgA responses, we posit that this is not a major pathway for responding to oral antigens.

After entry into GCs, proliferation and selection of high-affinity B cell clones to these model antigens in GALT occurs as it does in other lymphoid organs [33]. This requires antigen to be present within the GC, and thus, efficient transport from M cells through the SED into the GC must occur. Activated antigen-specific B cells themselves may possibly act as antigen carriers. After oral immunization of mice with NP–CT, transferred antigen-specific B cells with an activated phenotype are present in the SED even after a specific GC response is evident, and there is continuous migration of the SED B cells from the SED towards the GC during the response [22]. When PE-conjugated NP-hapten was injected into the intestinal lumen 10 days after oral NP–CT immunization, M cells transported NP–PE into SED and the migrating antigen-specific B cells bound to NP–PE, supporting a role for them in antigen-specific transport. There is also evidence for CCR6-mediated migration of B cells in the other direction, from the PPs towards the SED, during this response [21]. That B cells may migrate in both directions is supported by the clonal relationships that have been observed between B cells in SED and GCs [35]. Such bidirectional migration might allow for GC B cells of higher affinity to be recruited for antigen transport during the response, ensuring the continuous loading of antigens into the GC, provided that antigen is present in the gut to maintain continuous affinity maturation. In mouse lymph nodes and spleen, B cells have previously been found to act as nonspecific antigen carriers [44], and recently, antigen-specific transport was reported to also potentially occur in lymph nodes when B cells leave GCs [45]. Whether cells that migrate out of GCs to return within a short timeframe to GCs should be referred to as activated or early memory B cells, is mostly a question of semantics.

**Figure 3:**
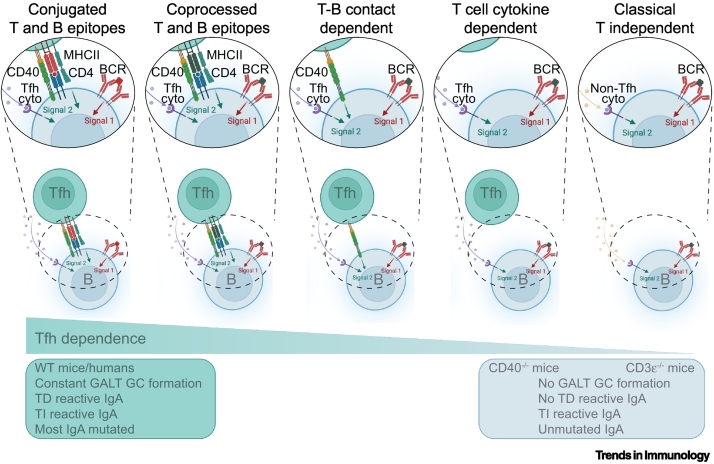
Potential mechanism supporting T-independent (TI) responses in gut-associated lymphoid tissue (GALT). Gut IgA binding to classical TI antigens carry mutated antibody variable regions, suggesting that responses against TI antigens in GALT differ from those in other tissues. While it is not exactly clear how this is mediated, different mechanisms can be imagined depending on the nature of the antigen**.** While TI antigens conjugated to proteins act as hapten carriers [33], many gut antigens can be present together, although not covalently linked (i.e., in intact bacteria and viruses). It is therefore possible that T cell epitopes can be presented by B cells that encounter them in the subepithelial dome (SED) [7,21]. Other responses may rely on noncognate interactions with T cells or triggering classical TI responses. Although gut IgA is produced in mice without any germinal centers (GCs) (because of lack of T cell functions), this IgA differs from that in wild-type (WT) mice (lacking variable region mutations); this must be taken into account in any model of TI responses in the gut [34,54]. Abbreviation: Tfh, T follicular helper.

**Figure 4:**
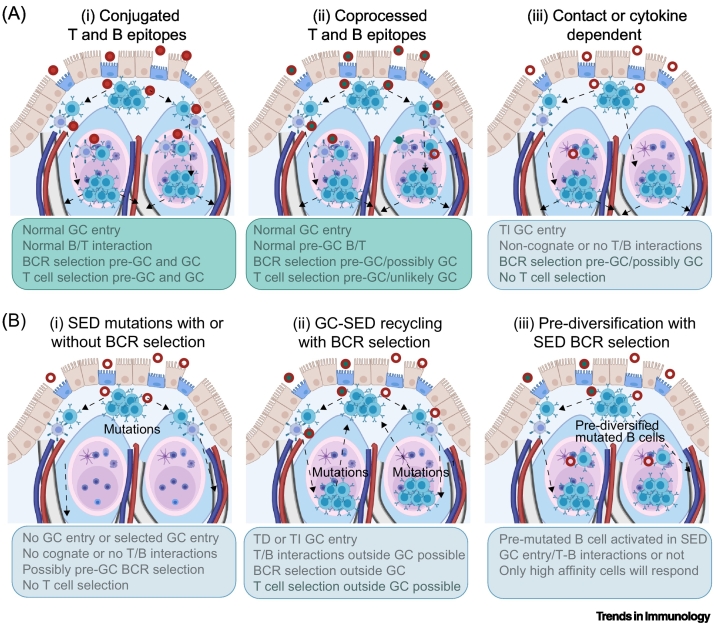
Potential consequences of antigen encounter in gut-associated lymphoid tissue (GALT). (A) For conjugated T-independent (TI) antigens, normal germinal center (GC) responses can occur (i) [33]. If TI and T-dependent (TD) antigens are coprocessed (ii), it is likely that B cells may present epitopes to pre-GC T cells in the subepithelial dome (SED), resulting in cognate interactions and GC entry, but unless intact antigens are transported together to the FDC network, classical GC selection involving T cell interactions cannot occur. For antigens lacking typical T cell epitopes (iii), it is still possible that B cells may enter into already existing GCs because signals required for GC formation and entry into pre-existing GC may differ. (B) Additional processes that might contribute to V region mutations and/or antigen selection in B cells reactive to TI antigens in GALT are mutations in proliferating cells in SED (i) [35,36], B cell receptor (BCR) mediated selection of high-affinity cells leaving GCs to enter the SED during a response (ii) [35,36] or activation of prediversified B cells (i.e., marginal zone B cells in humans or memory B cells with mutations that are not important for binding the original antigen); these may fortuitously be of higher affinity than cells carrying germ-line V regions (iii) [2]. These processes might not be mutually exclusive, but could all contribute.

On balance, we argue that it is likely that the initiation of B cell responses in GALT is most often a **T-dependent (TD)** process, although not generally involving crossdifferentiation of Th17 or Treg into Tfh cells. However, in a situation of antigen excess, it is plausible that B cells may enter GCs and undergo responses involving BCR receptor diversification, independent of T cells (Figure 3, Figure 4). This may be specifically important in the context of immune responses to microbiota since they are often covered with TI antigens to which IgA binds.

## IgA responses to microbiota

At first glance, recent mouse studies seemed to challenge the role of the microbiota in driving GALT B cells responses, because functional GCs are present even in germ-free mice ([46,47]. In these studies, highly expanded public clonotypes were found in PPs and mesenteric lymph nodes of germ-free mice. However, this should not be considered proof that non-antigen-driven processes dominate in GALT, but rather, as a demonstration of the disposition for GALT to form GCs. When microbiota were present, such responses were diluted out and more diverse B cell responses were observed. These responses differed between animals and were at least partly comprised of antibodies binding microbial antigens due to the presence of mutation-driven affinity maturation. Thus, while a strong drive to form GCs occurs in GALT, antigen-driven responses from the microbiota seem to take over and dominate in nonsterile conditions.

Many microbial antigens encountered in GALT, including lipopolysaccharides (LPSs) and polysaccharides, are TI antigens with repeating subunit structures. According to the classical view, such nonproteinaceous antigens encountered in the FAE and SED cannot be presented to T cells on MHC II and thus, B cells should not acquire cognate help to respond to them [48]. Despite this, IgA-secreting plasma cells that are specific for bacterial glycans are common [47,49., 50., 51.]. Proteins that can drive an extrafollicular response and IgA switch independently from CD40, such as a proliferation inducing ligan (APRIL), are important for the initiation of TI responses [34,52,53]. However, such responses still appear to be restricted to GALT and do not occur in the gut lamina propria [34]. Mechanisms that might support the production of IgA with specificity for nonprotein antigens in GALT are illustrated in Figure 3, Figure 4.

Despite specificity for TI antigens, IgA antibodies secreted by human and mouse intestinal plasma cells are encoded almost exclusively by immunoglobulin genes that have variable regions mutated by **somatic hypermutation (SHM)** [50,54]. Importantly, these somatic mutations enhance target binding, reflecting **affinity maturation** [50]. In addition, the acquisition of high mutation loads coupled with affinity selection are only known to occur in GCs, although low levels of mutations and IgA class switch can be observed in patients with CD40–CD40L deficiency, suggesting that our knowledge of this process remains incomplete [12,55,56]. Since GC development is conventionally dependent on cognate B cell interactions involving CD40/CD40L binding, as well as with a protein component to the antigen in humans and mouse models, this development presents an apparent paradox that might be resolved if TI-antigen-activated B cells entered pre-existing GCs, as occurs in a context of antigen excess in the mouse models described above (Figure 3, Figure 4). Entry of B cells into pre-existing GCs in GALT has been documented in mice where affinity maturation ensues following GC entry, if antigen is present [33,35]. It is possible that high quantities of antigen that accumulate in human GALT support the affinity maturation of immigrant B cells that are activated by TI antigen, without the T cell help that is normally required for B cell survival in GCs [57]. Supporting such a scenario, IgA responses can show **cross-species reactivity** [58], which would be related more to an abundance of antigen compared to the presence of species-specific antigens.

There is ample evidence that microbial antigens are a major factor driving GC responses in GALT, and that these responses mimic those in other lymphoid tissues with ongoing hypermutation and selection of high affinity B cells. However, the presence of large quantities of TI antigen from the gut microbiota and from already formed GCs, appears to create a unique environment where B cells reacting to TI antigens can both mutate their V regions and be selected for higher affinities. Thus, in this respect, GALT seem to differ from what is observed in other secondary lymphoid organs.

## GALT and systemic B cell development

The availability of microbial TI antigens in the gut also makes GALT a site that could preselect B cells that can bind to polysaccharide antigens systemically. Some species, including humans, are referred to as **GALT species**, in which GALT contributes to the creation of systemic B cell immunity [59]. The distinctive features of human GALT supporting this are summarized in Box 2.

Box 2 **Features of human GALT that can support its unique activities**

**Antigen acquisition**

The GALT does not have afferent lymphatics but receives particulate antigen via the specialized FAE that contains M cells, or by direct antigen sampling via dendritic cells [7,14].

**Antigen identity**

Particulate antigens sampled by GALT are predominantly bacterially derived from the microbiota in the gut lumen [17,19].

**B cell proximity to the microbiota**

B cells are present in intraepithelial locations close to the gut lumen [18,20,23]. This ensures that the expressed BCR has the potential to make direct contact with native antigen, to which many intestinal antibodies are directed. Most B cells in intraepithelial locations also express the inhibitory receptor FcRL4 that may also be key in modulating the B cell response to a strong BCR-derived signal [23,82].

**LysoDCs in the SED expressing microbicides and DNASE1L3**

The sampling of bacteria from the gut lumen can bring live bacteria inside the host. Key to maintaining host sterility is the LysoDC subset that sits between the epithelium containing B cells and the remaining lymphoid follicle. These cells can kill microbes including pathogens [14]. Microbial DNA is highly immunogenic, and DNASE1L3 secreted into the SED may be important for ensuring that microbial DNA does not elicit an anti-DNA response.

**TI B cell responses**

B cell responses in GALT including responses that are class switched to IgA can occur independently of cognate T cell help [12]. This is mediated in part by the cytokine APRIL that is produced by epithelial cells, by cells in the SED, and also within GCs [53].

**Life-long active GCs**

The chronic sampling of bacteria from the gut lumen is associated with the formation of GCs soon after birth [83]. GCs in this antigen-rich microenvironment appear to be sustained, the associated B cell responses can be extensive, and the immunoglobulin variable region genes can acquire very high loads of somatic mutations within large clones of plasma cell precursors [13].

**GALT GC entry in the absence of T cell help**

Evidence from animal models supports the concept that B cells can enter persisting GCs in situations of pre-existing antigen [33]. Antigens sampled from the gut lumen are likely to be present in GALT in excess. Propagation of B cells that recognize antigens expressed by many bacterial species might be favored [50].

Alt-text: Box 2

Recently reviewed, a role for GALT in the generation of antibody diversity was first defined in chickens by Max Cooper [59]. He removed the **bursa of Fabricius**, an anatomical structure closely associated with avian gut closely resembling GALT, and as a consequence, the birds could not mount antibody responses. Subsequently, the bursa in chickens was found to nurture developing cells, providing an environment for establishing receptor diversity in the B cell pool via **gene conversion** [59].

Although no bursal equivalent exists in mammals, GALT is involved in the generation of B cell diversity in sheep and rabbits via SHM, and gene conversion, respectively [59]. Although these key events occur *in utero*, in both cases, repertoire propagation by proliferation occurs once the gut is colonized by bacteria [59].

Evidence for an axis of shared cells between the spleen and GALT was identified in studies of humans with immunodeficiencies [60]. We now know that the role of GALT in postnatal human B cell development appears to be restricted to the maturation of innate-like **marginal zone B (MZB) cells** that develop over the first 2 years of life [59]. The MZ itself is a microanatomic region in GALT and spleen that can be occupied by memory B cells and MZB cells [18,61,62]. Studies of human immunodeficiencies and disease states confirm that memory B cells and innate-like MZBs are developmentally and functionally different [63., 64., 65.]. This is relevant because memory B cells also accumulate in the human spleen throughout life [66] and discriminating between memory B cells and MZBs can be difficult. Evidence supporting the role of GALT in human MZB development is summarized in Table 1.

**Table 1:** Evidence supporting the involvement of GALT in the maturation of human MZB cells

Observation	Refs
T2 transitional B cells in human blood with high expression of surface IgM (IgM^hi^) express α4β7 integrin that mediates homing into gut tissues by binding endothelial MAdCAM1.	[67]
MAdCAM1 is expressed by GALT endothelium and can thus mediate entry of cells expressing α4β7 integrin into GALT.	[9]
IgM^hi^ T2 B cells can be identified in GALT	[67]
IgM^hi^ T2 B cells express the gene encoding CD1c that is otherwise associated with marginal zone B (MZB) cells.	[67]
B cells expressing CD1c can be identified in fetal GALT. Fetal GALT microenvironment could potentially support the maturation of these cells into MZB cells.	[75]
T2 B cells in GALT are activated.	[85]
MZB cells can develop *in vitro* from marginal zone precursors expressing CD45RB by ligation of Notch2.	[69]
MZB cells and GC B cells isolated from GALT can be part of the same clone that shows evidence of proliferation and diversification in GALT.	[61]
B cells respond to a TI vaccine by rapid clonal expansion and production of plasmablasts; evidence of somatic hypermutation but no further somatic diversification within the clone.	[2]
B cells giving rise to cells that respond to a TI vaccine predominantly include MZB cells	[2]
Antibodies expressed by B cells responding to a TI vaccine recognize the gut microbiota. Acquired somatic mutations enhance the binding of capsular polysaccharides.	[2]
Both MZB and memory B cells can be identified in tissue sections of spleen and GALT. In each tissue, MZB are distributed closer to GCs than memory B cells.	[18,61,62]

Throughout life, human B cells continuously leave the bone marrow as T1 cells from which all other B cells subsequently develop. As B cells mature to T2 cells, they undergo a developmental bifurcation [67]. T2 cells expressing higher IgM (IgM^hi^) express integrin α4β7, which is associated with a cellular potential to extravasate at sites in the gut, including GALT, where they can be selectively identified. The receptor for integrin α4β7, MAdCAM-1, is expressed by endothelial cells in GALT and by splenic sinus-lining cells, consistent with a potential to share traffic in these tissues [8,9]. T2 cells expressing lower IgM (IgM^lo^) express CCR7, L-selectin, and IL4R, suggesting homing to peripheral, T-cell-rich tissues, capable of interacting with T cells [67].

IgM^hi^ T2 cells form a developmental continuum with MZBs that are also IgM^hi^ [67]. MZBs can be identified in GALT, locating close to GC [18,61]. Next-generation sequencing of human immunoglobulin genes identified B cell clones that included MZB and GC variants [61]. Lineage trees illustrating the relationship between B cells within a clone can be constructed by analyzing immunoglobulin heavy chain variable region sequences that are progressively mutated by SHM [68]. Such lineage trees, derived from GC and MZB variants within a clone, have shown hallmarks of MZB division and antibody diversification in GALT GC [61]. The GALT does not contain an abundance of MZBs; however, it is likely that these cells form a circulating continuum between the spleen (where they accumulate in the marginal zone) and the GALT [61,69]. Of note, the development of MZBs from **marginal zone precursor** B cells expressing a glycosylated variant of CD45RB is dependent on Notch2 signaling [70] and the distribution of Notch2 has been described in the GALT of rats [71].

Important for understanding the role of GALT in the development of MZB specificity and function, GALT exposure to bacteria equips MZBs with the capacity to rapidly respond to TI antigens administered systemically. Specifically, researchers tracked B cell responses to the TI Pneumovax vaccine that is composed of capsular polysaccharides from 23 pneumococcal serotypes using flow cytometry and antibody variable region sequencing [2]. The authors observed a rapid plasmablast response. Although the antibody variable region genes of the responding cells had previously undergone SHM, they did not further diversify upon challenge. Alignment of antibody variable region sequences with those of sorted B cells isolated prior to challenge, showed clonal relatives among the MZB population. The expressed antibodies had specificity for gut bacterial glycans consistent with prior exposure to gut microbes and SHM in GALT GC; this suggested that exposure to gut bacteria shaped MZB cell specificity [2].

From another angle, MZBs can be depleted in autoimmune diseases such as SLE [67]. Indeed, disruption of GALT structure in histological sections has also been observed in SLE patients [72]. It is unclear whether these observations are linked and whether this represents a change in development or a deviation from normal mature cell function.

## Concluding remarks

The GALT is a structure with contrasting major roles that are each dependent on the microbiota and which are essential for good health. The GALT protects and stabilizes the gut microenvironment by producing IgA plasma cell precursors. This IgA can have cross-species reactivity; that is, the ability to bind the same target on different bacterial species. Paradoxically, IgA specific for TI glycan antigens can use highly mutated antibody variable region genes, a feature that depends on a GC microenvironment otherwise associated with TD antigens. We propose that this is facilitated by TI antigen-dependent entry of B cells into pre-existing GCs. However, why only a few antigens can trigger efficient GALT responses instead of tolerance is an important question, not least of which is the development of putative oral vaccines against microbial infections. Indeed, many questions remain (see Outstanding questions).

A contrasting function of GALT in some species is the provision of a microenvironment that B cells can depend on for aspects of their development. Recently, humans were added to the list of GALT species based on the maturation of innate-like MZBs in GALT, where they acquire specificity for bacterial glycans, including capsular polysaccharides.

While the importance of GALT in generating IgA plasma cells has been known for decades, recent advances show that this enigmatic lymphoid tissue has unique properties that facilitate its maintenance of intestinal homeostasis, and the capacity to rapidly respond to pathogens that are encountered systemically [2].

Outstanding questions Can human B cells that are activated by TI antigens enter human GALT GC? This is assumed to occur based on animal models, and because B cells and plasma cells with specificity for intestinal TI antigens exist, and the receptors are mutated by SHM. However, there is no direct evidence for this. Assuming this can occur, what are the factors restricting B cell entry, propagation, and selection?

Why are so few model antigens available for GALT stimulation following oral challenge? While CT has been a valuable model oral antigen that can also function as an effective carrier of NP hapten, few other models are available. This is not for want of trying; the salient features of an effective oral antigen remain unclear.

How are GALT GCs initially established and does this vary along the GI tract? GALT GCs are initiated early in life, conventionally from activated B cells with cognate support from T cells. How is this achieved? Assuming T cells are involved, which T cells are these?

What is the relationship between GALT sites throughout the GI tract and what regulates it? We know that biases in factors that regulate plasmablast recruitment and also biases in IgA subclass distribution exist between the colon and ileum. Exactly how this is regulated remains unclear.

What is the function of FcRL4 that is expressed by human intraepithelial B cells in health? We know that FcRL4 is an inhibitory receptor that binds aggregated IgA, but its relevance in normal physiology remains obscure.

What directs the lineage split at the T2 stage of human B cell differentiation? A lineage split generates precursors of MZBs with gut homing properties. What regulates this? What happens in SLE to cause distortion of the B cell profile with MZ plasmablasts and MZB loss?

What is the relationship between GALT and splenic MZBs in humans throughout life? While evidence for this relationship is now strong, it is unclear whether this is more dominant in childhood (enriching the establishment of MZB repertoires) or adulthood.

What is the relationship between human MZBs and protection of the lung from pneumococcal disease? A major function of MZBs is the recognition of TI antigens including pneumococcal polysaccharides and protection from pneumococcal pneumonia. Thus, a B cell subset that matures in GALT and occupies the spleen can protect the lung. How the connection with the lung occurs is clinically important but not understood.

Alt-text: Outstanding questions

## Glossary

**Affinity maturation**: term used for SHM followed by selection of high affinity antibody variants during the GC response.
**Bursa of Fabricius**: lymphoid organ associated with the chicken gut where B cells develop.
**Cholera toxin (CT)**: antigen capable of driving potent immune responses in GALT, even when administered orally; also an adjuvant.
**Cross-species reactivity**: recognition of bacterial antigens shared across multiple bacterial species.
**Fc receptor like 4 (FcRL4)**: inhibitory receptor for aggregated IgA expressed by B cells within and adjacent to the FAE.
**Follicle-associated epithelium (FAE)**: epithelium overlying the lymphoid tissue; forms a barrier between the lymphoid tissue and the microbiota.
**GALT species**: species including chicken, sheep, rabbits, and humans that depend on the GALT and microbiota for certain aspects of B cell development.
**Gene conversion**: diversification process of a limited antibody variable gene repertoire in which antibody variable gene sequences derived from upstream pseudogenes replace homologous sequences in the original gene rearrangement.
**Germinal center (GC)**: microanatomical niche where B cells divide and undergo SHM and selection for specificity.
**Gut-associated lymphoid tissue (GALT)**: organized lymphoid tissue distributed adjacent to FAE in foci along the mammalian gastrointestinal tract.
**LysoDCs**: DCs in the SED that express microbicides and DNASE1L3 responsible for killing translocated microbes.
**Marginal zone B (MZB) cells**: innate-like B cells that mature in GALT in humans and that mediate immune responses to TI antigens including pneumococcal polysaccharides.
**Marginal zone precursors**: cells resembling naive B cells in their lack of CD27 and expression of IgM and IgD. Like naive B cells, they express the ABCB1 transporter but unlike naive B cells, they express the glycosylated form of CD45RB.
**Microfold (M) cells**: actively sample particulate antigens from the gut lumen and transport them to the lymphoid tissue below.
**Peyer’s patches (PPs)**: clusters of GALT in the terminal ileum.
**Regulatory T (Treg) cells**: specialized immunosuppressive CD4^+^ T cells that express the Foxp3 transcription factor.
**Somatic hypermutation (SHM)**: process of introducing mutations to antibody variable regions genes during B cell division, usually restricted to B cells in GCs.
**Subepithelial dome (SED)**: region beneath the epithelium where initial antigen encounter and also antigen destruction occur.
**T follicular helper (Tfh) cells**: specialized CD4^+^ T cell subsets that provide help to B cells; essential for GC formation.
**T helper 17 (Th17) cells**: CD4^+^ subset characterized by the production of IL-17 and expression of transcription factor RORγ; promote an immune response to extracellular pathogens.
**T-dependent (TD) B cell immune responses**: antibody responses to protein antigens that are dependent on cognate interactions between antigen-specific T cells via their TCR with peptides from the protein presented on MHC II by B cells.
**T-independent (TI) B cell immune responses**: responses to antigens that in a physiological context usually have a carbohydrate component and repeating subunit structures.
**Time of flight (ToF) techniques**: methods that use panels of rare earth metal-tagged antibodies to label cells either in suspensions (CyTOF) or in tissue sections (imaging mass cytometry). The metals are detected following laser ablation by mass cytometry.
**Toll-like receptors (TLRs)**: receptors of the innate immune system that detect pathogen associated molecular patterns.
